# On the dynamics and control of a squirrel locking its head/eyes toward a fixed spot for safe landing while its body is tumbling in air

**DOI:** 10.3389/frobt.2022.1030601

**Published:** 2022-11-24

**Authors:** Tianqi Ma, Tao Zhang, Ou Ma

**Affiliations:** ^1^ Department of Automation, Tsinghua University, Beijing, China; ^2^ Department of Aerospace and Engineering Mechanics, University of Cincinnati, Cincinnati, OH, United States

**Keywords:** squirrel flight behavior, righting reflex, body tumbling, attitude control, reinforcement learning

## Abstract

An arboreal mammal such as a squirrel can amazingly lock its head (and thus eyes) toward a fixed spot for safe landing while its body is tumbling in air after unexpectedly being thrown into air. Such an impressive ability of body motion control of squirrels has been shown in a recent YouTube video, which has amazed public with over 100 million views. In the video, a squirrel attracted to food crawled onto an ejection device and was unknowingly ejected into air by the device. During the resulting projectile flight, the squirrel managed to quickly turn its head (eyes) toward and then keeps staring at the landing spot until it safely landed on feet. Understanding the underline dynamics and how the squirrel does this behavior can inspire robotics researchers to develop bio-inspired control strategies for challenging robotic operations such as hopping/jumping robots operating in an unstructured environment. To study this problem, we implemented a 2D multibody dynamics model, which simulated the dynamic motion behavior of the main body segments of a squirrel in a vertical motion plane. The inevitable physical contact between the body segments is also modeled and simulated. Then, we introduced two motion control methods aiming at locking the body representing the head of the squirrel toward a globally fixed spot while the other body segments of the squirrel were undergoing a general 2D rotation and translation. One of the control methods is a conventional proportional-derivative (PD) controller, and the other is a reinforcement learning (RL)-based controller. Our simulation-based experiment shows that both controllers can achieve the intended control goal, quickly turning and then locking the head toward a globally fixed spot under any feasible initial motion conditions. In comparison, the RL-based method is more robust against random noise in sensor data and also more robust under unexpected initial conditions.

## 1 Introduction

**FIGURE 1:**
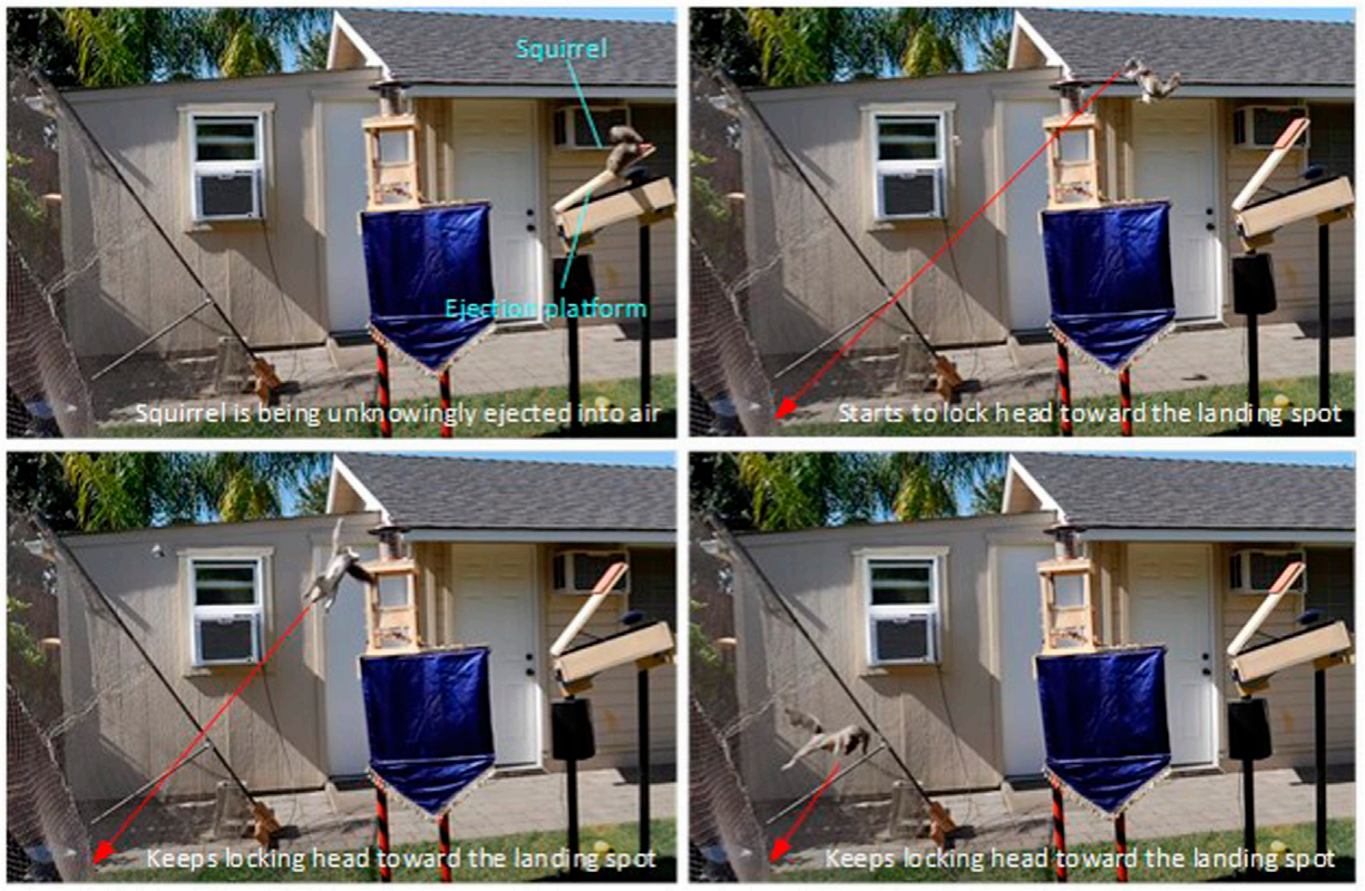
Squirrel is able to lock its head (and eyes) toward its landing spot while the rest of its body is tumbling after unknowingly ejected into air by the ejection platform [snapshots from the video (Rober, 2020)].

The righting reflex of animals is the ability to correct their body posture (orientation) from an abnormal posture on emergency. A well-known example is that when a cat falls from a high position, it can always manage to change its body orientation and land right on its feet even if it falls from an upside-down posture (Kane and Scher, 1969). A similar phenomenon has also been observed on many other species, such as lizards (Sinervo and Losos, 1991; Schlesinger et al., 1993) and rats (Laouris et al., 1990). Such a mid-air body posture control ability helps animals reduce the risk of body injury from a free fall. This kind of amazing body posture control (attitude control) behavior of cats and other animals is difficult to understand due to the conservation of momentum, which tells us that in a free fall, nobody (regardless a human or animal) can change their angular momentum by moving body segments (head, limbs, tail, *etc.*) because all the efforts generate internal forces/moments only. Therefore, the well-known cat free-fall problem has motivated many research efforts in the past, and the findings have been well documented (Fernandes et al., 1993; Arabyan and Tsai, 1998; Weng and Nishimura, 2000; Xu et al., 2012).Arboreal mammals such as squirrels face higher risk of unexpected falls than cats (Young and Chadwell, 2020) due to their natural habitats and aggressive activities. Squirrels are hardly injured by falls from height because of their impressive self-control ability of their body posture. In a highly popular YouTube video (Rober, 2020), squirrels were lured by food onto an ejection device and they were suddenly ejected into air (as shown in Figure 1). Obviously, the ejection caused their bodies to tumble (general 3D rotation) and free fall in air. Amazingly, an ejected squirrel could quickly (within 0.1 s) turn its head toward where it would land and then lock its head (thus eyes) to that direction for staring at the landing spot, while the rest of its body continued tumbling in air. At the end, the squirrel always landed at it stared landing spot with its feet touching down first. The squirrels’ such amazing behavior of locking head toward a fixed spot understandingly is for better situation awareness and safe landing. However, the question of how they can achieve that capability from dynamics and control perspective has not been studied in the literature. Our research is to address this problem.

Understanding how animals control their body postures in the absence of external forces (except gravity) is not only driven by our scientific curiosity but also highly motivated by engineering needs, especially robotics. Understandably, the principles of animals controlling their body postures using their body part movements can be applied to robots for achieving similar or even better behavior. In this regard, research works have been conducted (Shuster et al., 1993; Tsiotras, 1996). For example, controlling body posture without external force is necessary in some special task environments such as space, disaster scene (Gonzalez et al., 2020), and hopping robots (Yim et al., 2020). Damage by falls (Cameron and Arkin, 1992) could also be prevented to extend servicing and lifespan of robots (Bingham et al., 2014).

The research inspired by animal motions to control body postures can be divided into the following three aspects:

1) Using tails to control their body orientation: This kind of research particularly focuses on imitating lizards (Jusufi et al., 2010; Libby et al., 2012; Clark et al., 2021) and geckos (Jusufi et al., 2008; Siddall et al., 2021). These kinds of arboreal animals often have a tail with large moment of inertia and a body without obvious internal motions. Therefore, the lizards/geckos can reorient or stabilize the body by moving the tail (Wenger et al., 2016) and they can be simply modeled as two linked rigid bodies. The tail is known to take a role in controlling the pitch angle of the lizards/geckos in the air for landing (Libby et al., 2012; Siddall et al., 2021), air-righting like a cat (Jusufi et al., 2010, 2008). For gliding lizards, tails also help adjusting the angle of attack to improve both glide distance and stability (Clark et al., 2021). Similar work on squirrels (Fukushima et al., 2021) also explained the stability using the motion of tails in unexpected falls. Special tails were added to help robot jumping (Zhao et al., 2013), insect-sized robot achieving more rapid orientation (Singh et al., 2019), and more complicated tails (e.g., a three-segment prototype (Liu and Ben-Tzvi, 2020)) and soft tails (Butt et al., 2021) were designed to perform as real tails, which is not rigid in reality.
2) Using the whole body to reorient body posture (or attitude) (Kane and Scher, 1969; Sadati and Meghdari, 2017; Liu et al., 2020; Yim et al., 2020): In this kind of research, models do not need a special part that absorbs extra angular momentum, instead, the control strategy is to redistribute the angular momentum to different parts of the body to achieve different goals. For example, cats do not necessarily need their tails to change their body orientation in air (Fredrickson, 1989). Hence, the control of a cat-like robot needs to consider all the joints of its body (Arabyan and Tsai, 1998). Flying snakes slide with aerial undulation to increase performance and could be another inspiration for dynamic flying robots (Yeaton et al., 2020).
3) Using the aerodynamic effect on the special structure of the body (Li et al., 2016; Norby et al., 2021): Since the motion is in air, robots can imitate the gliding principle of animals, such as flying squirrels. Using the aerodynamic effect wisely can control body attitude and reduce the energy consumption.

The squirrel behavior shown in the study proposed by Rober (2020) inspired this work. We focus on the control of turning and locking the head of a squirrel toward the landing spot when it is initially thrown into air and remains tumbling in air. We study the possibility of using joint motion control (like a robot) to regulate the head orientation. For easy understanding of the dynamics, we use planar multibody dynamics (in the pitch plane) to model a squirrel. Such a 2D modeling approach has been widely applied by other researchers for other bio-inspired robotics problems, such as unmanned aerial vehicle (Bouabdallah and Siegwart, 2007; Yilmaz et al., 2019; Zamora et al., 2020), and other tasks, such as trajectory tracking (Castillo-Zamora et al., 2019), grasping objects (Kobilarov, 2014; Zhao et al., 2018), and sports (table tennis) (Shi et al., 2019).

In the squirrel problem, there are three features in the motion of sudden ejection of the squirrel:

1) The ejection occurs suddenly so that there is no preparation time
2) The initial posture (initial motion conditions) is random
3) The flight duration is short, and thus, a timely response is required

These factors contribute to the challenge of the investigated motion control. In our study, we apply reinforcement learning (RL) technology to train a neural network-based control algorithm to deal with the non-linear dynamics and control problem just as those having been tried in different bio-inspired robots (Dooraki and Lee, 2019; Li et al., 2019; Kamali et al., 2020). At the same time, we also apply a traditional PD controller along with an optimal trajectory planning approach as an alternative and baseline for comparison.

We believe that aerodynamics also plays a role in a real squirrel’s flight behavior because its furry tail can cause enough air drag (external force) to change its body momentum. However, in this study we are investigating the dynamics and control only in the case of conservation of momentum without external force. We know that animals can achieve their desired motion behavior by only controlling the relative motions of their body segments and limbs without air drag on tails. For example, even a tailless cat can land on its four feet after it is dropped from an upside-down configuration (Fredrickson, 1989). We leave the investigation of the complex aerodynamic modeling and analysis of the furry tails to future research.

The rest of this article is organized as follows: In Section 2, the dynamics formulation of the model is introduced. Section 3 describes the RL-based method and PD controller. The simulation environment and results are in Section 4. Finally, the article is concluded with a discussion in Section 5.

## 2 Dynamics of the 2D multilink model

### 2.1 Dynamics

**FIGURE 2:**
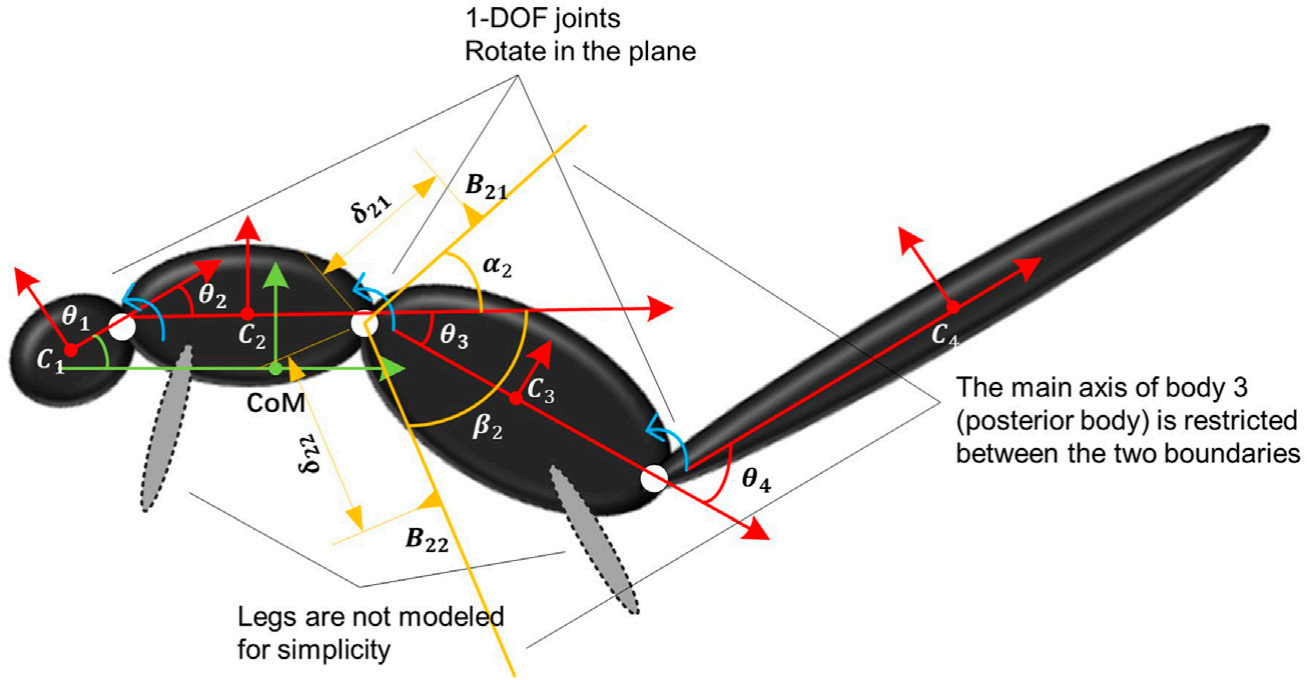
Kinematic notation of the multibody system and contact envelope between body *i*−1 and *i* with *i* = 3.

In general, we can model the squirrel with *N* rigid bodies connected with each other. *O*_*i*_, *O*_*C*_, and *O*_*W*_ are the origins of the coordinate frames attached to the *i*th body, the center of mass (CoM) of the multibody system, and the inertial frame, respectively. These symbols are also used to represent their associated coordinate frames. Let *θ*_1_ be the angle between the major axis (defined as the *y* axis) of the first body and the *y* axis of frame *O*_*C*_. Then, *θ*_*i*_ (*i* = 2, … , *N*) is defined as the angle between the major axis of the *i*th and *i* − 1th body. The configuration of the system can be determined by $\theta ={\left[\theta_{1},\theta_{2},\ldots ,\theta_{N}\right]}^{\text{T}}\in R^{N}$ . A multibody rigid model of a squirrel with *N* = 4 is shown in Figure 2. Next, we can derive the dynamics of the multibody system (Featherstone, 1984) in frame *O*_*C*_, following Lagrange’s equations of the second kind. The dynamic equation of the system is

$$M\left(\theta \right)\ddot{\theta }+C\left(\theta ,\dot{\theta }\right)\dot{\theta }={\left[0,\tau +\tau_{\text{c}}\right]}^{\text{T}},$$ (1)

where **M**(***θ***) is the positive-definite inertia matrix, which is determined by the configuration ***θ***, $C\left(\theta ,\dot{\theta }\right)$ is the Coriolis matrix, which is determined by the configuration ***θ*** and its derivative $\dot{\theta }$ . We omit the gravity force term in the left-hand side since Eq. 1 is derived in the frame *O*_*C*_. In fact, gravity only has influence on the position, velocity of CoM, and thus the duration of the flight, but when we focus on the configuration ***θ*** only, the gravity does not have influence. In simulation, we can calcuate ***θ*** and the position of CoM, respectively and separately, as shown in Section 4.1.

Since we could only control the extra inner wrench on each joint, the first element of the right-hand side of Eq. 1 should be zero and the rest of the elements $\tau \in R^{N-1}$ are the torques applied to the *N* − 1 joints, i.e., the joint control torques. ***τ***_c_ is the vector of the generalized torques caused by collision between body segments and it is non-zero only if such a collision occurs. Since we assume that body segment collisions are intermittent, in our simulation we calculate the velocities right before and after a collision rather than the specific ***τ***_c_, which we discuss in detail in Section 2.2. Considering that the CoM of the system moves only under the action of gravity, the motion of the system can also be determined in the frame *O*_*W*_.

The configuration ***θ*** (*t*) is determined by three different factors: 1) the joint control torques; 2) the coupling effect caused by the joint motion; and 3) the initial motion conditions. The second term is the main source of non-linearity. The third term determines the total linear and angular momentums of the system, which cannot be changed during the entire flight phase. In this study, we set $\dot{\theta_{1}}\left(0\right)=\pi /2$ rad/s and $\dot{\theta_{i}}\left(0\right)=0$ for *i* = 2, … , *N* (meaning that the squirrel rotates at *π*/2 rad/s at the beginning with respect to where it stands/sits). In 2D simulation, $\dot{\theta_{1}}\left(0\right)$ cannot be too large, otherwise, factor 3 mentioned previously determines the whole flight and make it very difficult to control. In this study, we particularly focus on joint control of the system to achieve the goal that the head (body 1) must be quickly turned to facing the landing spot after ejection and then lock toward the landing spot throughout the rest of the projectile flight.

### 2.2 Intermittent contact between the bodies

When a squirrel (or another animal) is unintentionally thrown into air leading to tumbling in flight, some of its body segments will intermittently contact with each other causing impact and bouncing of the contacting bodies during the flight. This physical phenomenon should be captured in the modeling for realistic simulation. To simulate this body segment contact behavior, we add a contact modeling constraint. In general, we consider the contact model between bodies *i* − 1 and *i* and show an example with *i* = 3 in Figure 2. The orange lines are the two physical boundaries of body *i* − 1 with angles *α*_*i*_ and *β*_*i*_, respectively. In this way, body *i* is restricted between the two boundaries, i.e., *θ*_*i*_ ∈ [ − *β*_*i*_, *α*_*i*_], *i* = 2, … , *N*.

To calculate the new motion state immediately after contact, we assume two contact points *B*_*i*1_ and *B*_*i*2_ on the two boundaries to determine the contact location. Let $\left|B_{i1}\right|=\delta_{i1}$ , $\left|B_{i2}\right|=\delta_{i2}$ , and *θ*_*i*_ = *α*_*i*_ when the impact occurs. The relative angular velocity of body *i* relative to *i* − 1 is $\dot{\theta_{i}}$ and the relative velocity of *B*_*i*1_ on body *i* relative to *i* − 1 right before impact is

$$\Delta v_{B_{i1}}={\left[-{\dot{\theta }}_{i}\delta_{i1}\sin\left(\theta_{i}\right),{\dot{\theta }}_{i}\delta_{i1}\cos\left(\theta_{i}\right)\right]}^{\text{T}}.$$ (2)

Then, the projected relative velocity of *B*_*i*1_ on body *i* relative to *i* − 1 in the impact direction is

$$\Delta v_{B_{i1}}^{\text{P}}=R\left(\overset{i}{\underset{j=1}{\sum }}\theta_{j}\right)\Delta v_{B_{i1}}={\left[0,{\dot{\theta }}_{i}\delta_{i1}\right]}^{\text{T}},$$ (3)

where **R** (⋅) represents the 2D rotation matrix. Right after the impact, the relative angular velocity changes to ${\dot{\theta }}_{i}^{\prime }$ , and the separation velocity at *B*_*i*1_ is

$$\Delta {v_{B_{i1}}^{\text{P}}}^{\prime }={\left[0,{\dot{\theta }}_{i}^{\prime }\delta_{i1}\right]}^{\text{T}}.$$ (4)

Assuming the coefficient of restitution of the impact is *k*,

$$-k{\dot{\theta }}_{i}\delta_{i1}={\dot{\theta }}_{i}^{\prime }\delta_{i1}.$$ (5)

It is easy to figure out that *δ*_*i*1_ (i.e., the position of each contact point) has no influence on the angular velocity after impact, i.e.,

$$-k{\dot{\theta }}_{i}={\dot{\theta }}_{i}^{\prime }.$$ (6)

In addition to Eq. 6, the conservation law of angular momentum in the frame *O*_*C*_ also needs to be satisfied. In frame *O*_*C*_, the angular momentum of the system is

$$H=\overset{N}{\underset{i=1}{\sum }}J_{i}\dot{\theta_{i}}+y_{ci}m_{i}{\dot{z}}_{ci}-z_{ci}m_{i}{\dot{y}}_{ci},$$ (7)

where *m*_*i*_, *J*_*i*_, *y*_*ci*_, and *z*_*ci*_ are the mass, momentum of inertia, and *y*- and *z*-axis coordinates of the CoM of the *i*th body, respectively. Since the coordinates of each CoM are determined by the configuration ***θ***, *H* can be rewritten as a function of ***θ*** and $\dot{\theta }$ and the function is linear with respect to $\dot{\theta }$ , namely, $H=\tilde{J}{\left(\theta \right)}^{\text{T}}\dot{\theta }$ , where $\tilde{J}$ can be regarded as an “equivalent” inertia tensor corresponding to $\dot{\theta }$ . The conservation law of angular momentum on the impact between bodies *i* − 1 and *i* is

$$\overset{i+1}{\underset{j=i-1}{\sum }}{\tilde{J}}_{j}\overset{j}{\underset{k=i-1}{\sum }}{\dot{\theta }}_{k}=\overset{i+1}{\underset{j=i-1}{\sum }}{\tilde{J}}_{j}\overset{j}{\underset{k=i-1}{\sum }}{\dot{\theta }}_{k}^{\prime }.$$ (8)

Additionally, the relative angular velocity of body *i* + 1 with respect to that of body *i* should be the same right before and after the impact, namely,

$$\overset{i+1}{\underset{j=i-1}{\sum }}{\dot{\theta }}_{j}=\overset{i+1}{\underset{j=i-1}{\sum }}{\dot{\theta }}_{j}^{\prime }.$$ (9)

To summarize, Eqs 6, 8, 9 form the equations relating the angular velocities right before and after the impact. If more than two bodies simultaneously collide, we can use similar equations to calculate. For example, if bodies *i* − 1, *i*, and *i* + 1 collide at the same time, then the equations

$$\begin{array}{cc} & \overset{i+2}{\underset{j=i-1}{\sum }}{\tilde{J}}_{j}\overset{j}{\underset{k=i-1}{\sum }}{\dot{\theta }}_{k}=\overset{i+2}{\underset{j=i-1}{\sum }}{\tilde{J}}_{j}\overset{j}{\underset{k=i-1}{\sum }}{\dot{\theta }}_{k}^{\prime }\\ & \overset{i+2}{\underset{j=i-1}{\sum }}{\dot{\theta }}_{j}=\overset{i+2}{\underset{j=i-1}{\sum }}{\dot{\theta }}_{j}^{\prime }\\ & -k{\dot{\theta }}_{i}={\dot{\theta }}_{i}^{\prime }\\ & -k{\dot{\theta }}_{i+1}={\dot{\theta }}_{i+1}^{\prime } \end{array}$$ (10)

can be used to determine the motion state right after the impact.

## 3 Control method

In this section, we develop the control methodology of locking the first body of the multibody system (i.e., the head of the squirrel) toward the landing point during the flight phase by applying joint control efforts. We apply two different control methods to achieve the control goal: 1) trajectory planning in addition to a PD feedback control law and 2) an RL-based control policy. The first method is a traditional control approach while the second is a machine learning-based approach.

### 3.1 Trajectory planning for a PD controller

**FIGURE 3:**
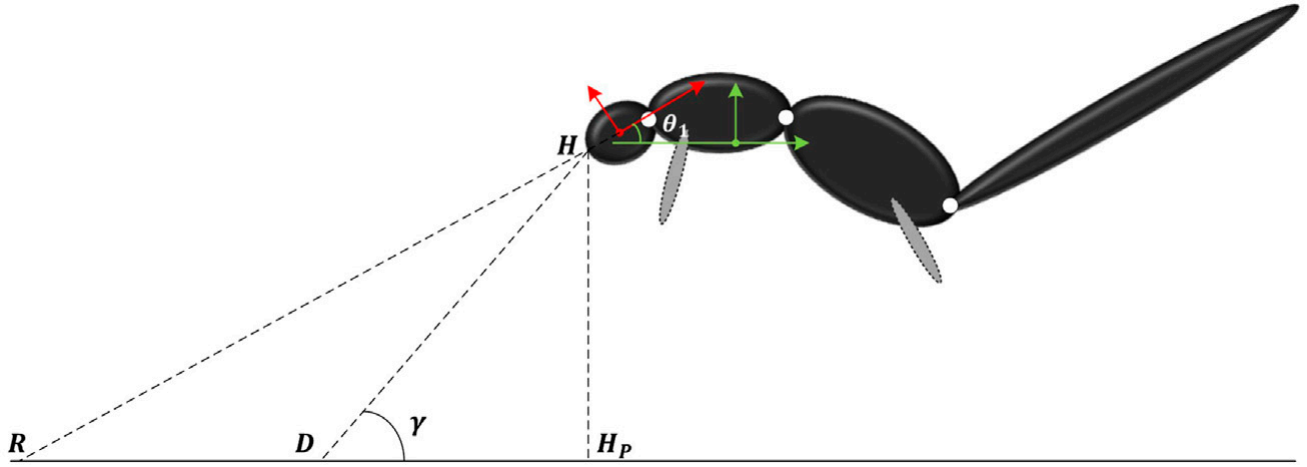
Parameters to optimize during the flight phase.

A PD controller needs a pre-planned motion trajectory as a reference to determine the position and velocity errors as the input to the PD control law. Therefore, we need to calculate a reference trajectory that represents the squirrel’s motion behavior. It is clear that the CoM of the squirrel follows the projectile motion trajectory determined by the gravitation and squirrel’s initial conditions. However, the relative motion of the individual bodies or joints of the squirrel will be governed by the multibody dynamics and conservation of angular momentum. As shown in Section 2, the dynamics of the system is non-linear, which makes it difficult to derive an analytic solution of the dynamic system. Hence, we choose to solve a set of optimization problems for the trajectory planning.Assume *D* is the fixed landing point on the ground, *R* is the moving looking point, *H* is the head point, *H*_*p*_ is on the ground, and *HH*_*p*_ is parallel to the *z* axis of the frame *O*_*W*_, as shown in Figure 3. To ensure the head (Body 1) to look toward the landing point *D* at (*y*_*l*_, 0) in frame *O*_*W*_, *θ*_1_ should be equal to *γ* in Figure 3, which is the angle between *HD* (the line between the head point and landing point) and the *y* axis of the frame *O*_*W*_. Then, the optimization objective is

$$\underset{\theta }{\min}\frac{1}{2}{\left(\tan\gamma -\tan\theta_{1}\right)}^{2}.$$ (11)

To avoid singularity when *θ*_1_ = *π*/2 and |*DH*_*p*_| = 0, we use the following optimization objective instead:

$$\underset{\theta }{\min}\frac{1}{2}{\left(|HH_{p}|\cos\theta_{1}-|DH_{p}|\sin\theta_{1}\right)}^{2}.$$ (12)

During the flight, the total angular momentum of the system in the frame *O*_*C*_ in Eq. 7 should be constant. Assuming that the initial angular momentum is *H*_0_ and the configuration of the system at time = *t* is ***θ*** (*t*), then from time = *s* to time = *s* + *δs*, where *δs* is a short time period

$$\int_{s}^{s+\delta s}{\tilde{J}\left(\theta \left(t\right)\right)}^{\text{T}}\dot{\theta }\left(t\right)\;\text{d}t=H_{0}\delta s.$$ (13)

If $\tilde{J}\left(\theta \left(t\right)\right)$ remains almost constant, i.e., $\tilde{J}\left(\theta \left(s\right)\right)$ , during *δs*, then

$${\tilde{J}\left(\theta \left(s\right)\right)}^{\text{T}}\left(\theta \left(s+\delta s\right)-\theta \left(s\right)\right)\approx H_{0}\delta s.$$ (14)

However, during simulation, we find that if we set *D* as the landing point from the beginning, the configuration solution of the system at time = 0 will be quite different from the initial configuration, and Eq. 14 is no longer satisfied. To solve this problem, we introduce a moving point *D*_*f*_ on the ground as a pseudo landing point at (*y*_*pl*_(*t*), 0), whose y position is defined as

$$y_{pl}\left(t\right)=\max\left(y_{l},y_{ml}\left(t\right)\right),$$ (15)

where

$$y_{ml}\left(t\right)=y_{R0}-\frac{g\left(y_{R0}-y_{l}\right)}{0.2\times \left({\dot{y}}_{\mathrm{CoM}}+\sqrt{{{\dot{y}}_{\mathrm{CoM}}}^{2}+2gz_{\mathrm{CoM}}}\right)}t,$$ (16)

where *y*_*R*0_ is the initial y coordinate of *R* in Figure 3 and *g* = 9.81 *m*/*s* ^2^ is the gravity acceleration. In this way, *D*_*f*_ moves from the initial position of *R* to the target point *D* in the process, and thus, we have Algorithm 1 for calculating the reference trajectory.

Algorithm 1 Calculation of the reference trajectory.

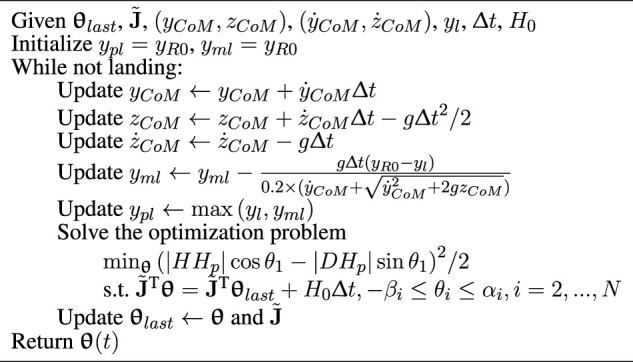

**FIGURE 4:**
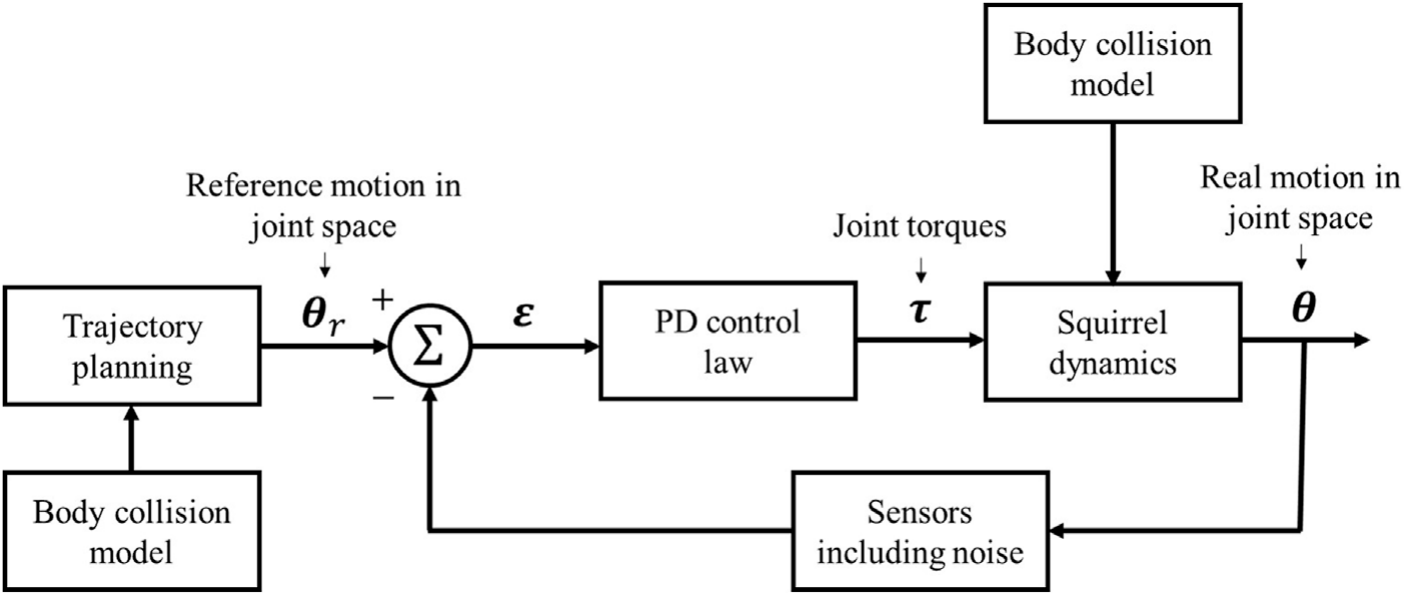
Diagram of the joint PD control process.

Suppose a reference trajectory ***θ***_*r*_(*t*) is obtained from Algorithm 1, then we propose a PD controller to allow the system to track the reference trajectory in the way, as shown in Figure 4. Suppose $\tilde{\theta }={\left[\theta_{2},\ldots ,\theta_{N}\right]}^{\text{T}}$ and ${\tilde{\theta }}_{r}={\left[\theta_{2r},\ldots ,\theta_{Nr}\right]}^{\text{T}}$ , the tracking error is calculated as

$$\varepsilon =2\left({\tilde{\theta }}_{r}-\tilde{\theta }\right)/\pi .$$ (17)

We choose the control gains of the PD controller on each joint separately. Let joint *i* be the joint between body *i* − 1 and *i* and *K*_*pi*_, *K*_*di*_ be the corresponding control gains, then the output of the PD control law is defined as

$$\tau =K_{p}\varepsilon +K_{d}\frac{\text{d}\varepsilon }{\text{d}t}\approx K_{p}\varepsilon +K_{d}\frac{\varepsilon -\varepsilon_{\mathrm{last}}}{\Delta t},$$ (18)

where **K**_*p*_ = diag (*K*_*p*2_, *K*_*p*3_, … , *K*_*pN*_) and **K**_*d*_ = diag (*K*_*d*2_, *K*_*d*3_, … , *K*_*dN*_). Physically, ***τ*** is a vector whose components are the required joint control torques.

### 3.2 Reinforcement learning-based method

Conventional control methods, as the one described in Section 3.1, usually require trajectory planning, which is a difficult problem if one does not fully understand how a squirrel controls its body segments (i.e., joint motions) for a target motion behavior. In fact, trajectory planning can be avoided by applying reinforcement learning technology in the control solution for the squirrel to achieve an expected behavior. In this section, we apply the proximal policy optimization (PPO) algorithm (Schulman et al., 2017) as the RL method to train a control policy for the squirrel to achieve the desired behavior.

**FIGURE 5:**
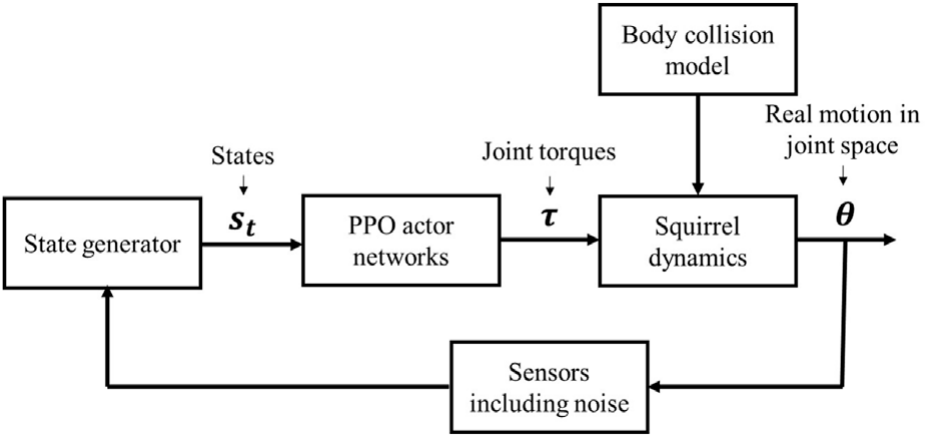
Diagram of the RL-based process.

PPO has an actor-critic architecture and works for both discrete and continuous action domains. It trains a policy to obtain the maximum expectation of total reward which we set before a training. Therefore, if we set the reward properly, the policy trained by PPO will implement specific functions. To access the desired policy *π*_Θ_ using the PPO2 algorithm (Schulman et al., 2017), the optimization objective L to maximize is defined as

$$\begin{array}{cc} L^{\Theta^{\prime }}\left(\theta \right)={\text{E}}_{\left(s_{t},a_{t}\right)∼\pi_{\Theta^{\prime }}} & \min\left[\frac{p_{\Theta }\left(a_{t}|s_{t}\right)}{p_{\Theta^{\prime }}\left(a_{t}|s_{t}\right)}A^{\Theta^{\prime }}\left(s_{t},a_{t}\right),\right.\\ & \left.\text{clip}\left(\frac{p_{\Theta }\left(a_{t}|s_{t}\right)}{p_{\Theta^{\prime }}\left(a_{t}|s_{t}\right)},1-\epsilon ,1+\epsilon \right)A^{\Theta^{\prime }}\left(s_{t},a_{t}\right)\right] \end{array},$$ (19)

where (*s*_*t*_, *a*_*t*_) represent the state and action of the agent at time = *t*; *π*_Θ′_ represents another policy with parameter Θ′ (usually similar to *π*_Θ_ by applying minimization and clip function); *p*_Θ_/*p*_Θ′_ represents corresponding probability; and *A* ^Θ^(*s*_*t*_, *a*_*t*_) represents the advantage function under state *s*_*t*_ and action *a*_*t*_ of policy *π*_Θ_. Usually, for an RL trajectory *τ* with length *T* (defined as a series of state-action pair (*s*_0_, *a*_1_, *s*_1_, … , *a*_*T*_, *s*_*T*_)), we set

$$A^{\Theta }\left(s_{t},a_{t}\right)=\overset{T}{\underset{t^{\prime }=t}{\sum }}\gamma^{t^{\prime }-t}r_{t^{\prime }}-\text{E}\left[R\left(\tau \right)\right],$$ (20)

where *r*_*t*′_ denotes the one-step reward at *t*′ in an RL trajectory *τ*; *γ* ∈ [0, 1) denotes the discount factor; and E [*R*(*τ*)] (approximated by a neural network) is the expectation of trajectory reward *R*(*τ*).The control method is based on the PPO2 algorithm, as shown in Figure 5. The vector **s**_*t*_ describes the current status of the system and it consists of the head coordinate in the frame *O*_*W*_, ***θ***, $\dot{\theta }$ , and the velocity of the CoM in the frame *O*_*W*_. The vector ***τ*** is the control torque applied on each joint and is sampled from a trainable normal distribution. For one-step reward, since a desired policy should 1) minimize the distance between point *R* and the landing point (or the pseudo landing point); 2) avoid high angular velocities; and 3) avoid high joint torques. The first objective is to lock the head (body 1) toward the landing point, while the other two are to reduce the kinetic energy. Thus, there are three different settings.

$$\begin{array}{cc} & r_{1}=0.1\text{exp}\left(-\left|y_{R}-y_{l}\right|\right)+0.01\text{exp}\left(-0.1\left\|\dot{\theta }\right\|\right)+0.001\text{exp}\left(-\left\|\tau \right\|\right)\\ & r_{2}=0.1\text{exp}\left(-\left|y_{R}-y_{l}\right|\right)-0.001\left\|\dot{\theta }\right\|-0.001\left\|\tau \right\|\\ & r_{3}=0.1\text{exp}\left(-\left|y_{R}-y_{pl}\right|\right)-0.001\left\|\dot{\theta }\right\|-0.001\left\|\tau \right\| \end{array}.$$ (21)

These three reward settings aim at minimizing the distance between point *D* (or *D*_*f*_) and *R*, the *l*_2_-norm of $\dot{\theta }$ and ***τ***, respectively. The difference among the reward settings also focuses on a smoother configuration trajectory from two aspects: 1) training with *D*_*f*_ instead of *D* and 2) different ways of penalty on $\dot{\theta }$ and ***τ***.

**FIGURE 6:**
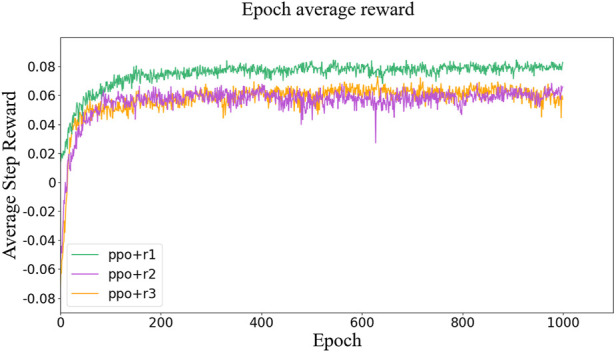
Values of the reward function over the training process.

We train control policies with the three rewards for 1,000 epochs with 2,048 timesteps in each epoch. The values of the reward function over the training process are shown in Figure 6, where the rewards all tended to saturate near end and little gain can be obtained from further training.

## 4 Simulation results and discussion

### 4.1 Simulation environment

The simulation environment was established on the Ubuntu 20.04 system with a 16 GB RAM and an Intel Core i9-9900kf CPU. We simulated the motion behavior by calculating and recording the motion for each time step using Python. The process is described in Algorithm 2. It used the Newmark-beta method (Newmark, 1959), as the method of numerical integration to solve Eq. 1 with time step Δ*t* = 0.001 s.

Algorithm 2 The process of the simulation.

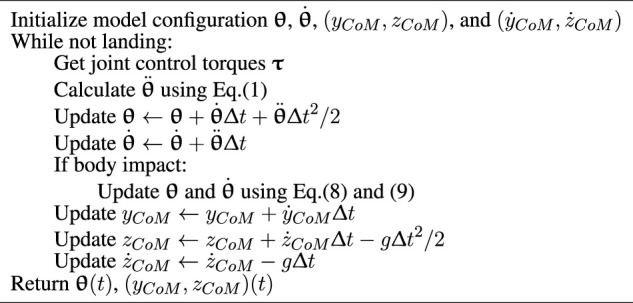

**FIGURE 7:**
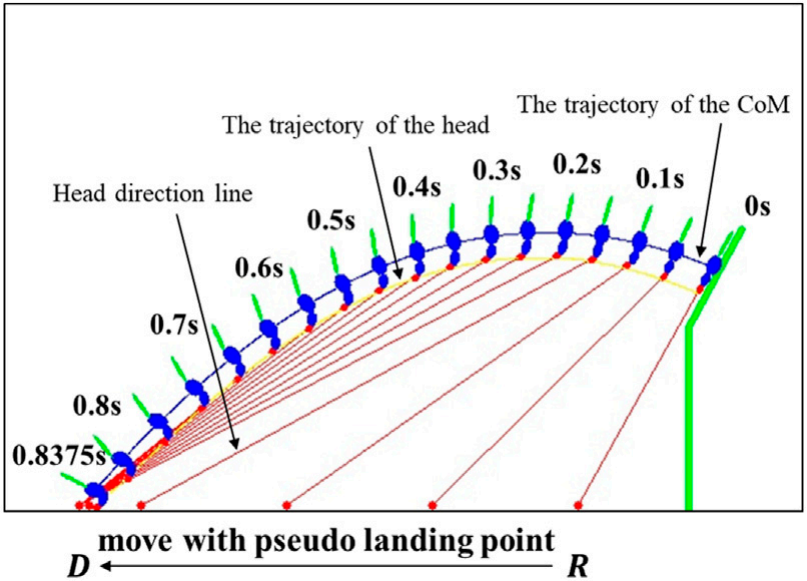
Reference trajectory calculated by Algorithm 1.

In the simulation, we set *N* = 4 and the four bodies are the head, upper body (chest portion of the body trunk), lower body (abdomen portion of the body trunk), and tail. The model parameters and the selected PD control gains are shown in Table 1.The platform ejecting the squirrel has an initial angular velocity of 4*π* rad/s, and thus the velocity of the CoM is determined by where the squirrel is initially located on the platform. However, once the squirrel detects the sudden movement of the platform, it will quickly move its body to adjust its body posture and thus its initial angular velocity can be different from that of the platform. In the simulation, we set the joint motion for the squirrel model as *π*/2 rad/s, as shown in Table 1. The reference trajectory generated based on Algorithm 1 is shown in Figure 7.

In the following results, the simulation has been repeated 100 times with or without random noise in the feedback information. The noise is defined as a Gaussian distribution of 0 mean and 0.05*A* standard deviation, where *A* is the maximal measuring range of an input variable (e.g., *θ*_4_ ∈ [ − 0.75*π*, 0.75*π*] then *A* = 0.75*π*). Therefore, both the nominal motion and noised motion (with the standard deviation of the error distribution) of the squirrel are shown in the following sections.

**TABLE 1:** Parameters of the model, kinematics, and PD controller.

Data item	Value	Data item	Value	Data item	Value	Data item	Value
*m*_1_	0.05 kg	*l*_4_	0.2 m	*k*	0	***K***_*p*_(Joint 1)	0.66
*m*_2_	0.35 kg	*b*_1_	0.05 m	*α*_2_	60°	***K***_*p*_(Joint 2)	0.55
*m*_3_	0.4 kg	*b*_2_	0.07 m	*α*_3_	90°	***K***_*p*_(Joint 3)	0.19
*m*_4_	0.2 kg	*b*_3_	0.1 m	*α*_4_	135°	***K***_*d*_ (Joint 1)	0.009
*l*_1_	0.06 m	*b*_4_	0.02 m	*β*_2_	90°	***K***_*d*_ (Joint 2)	0.011
*l*_2_	0.1 m	***θ*** (0)	[61°, 0°, 0°, 0°]	*β*_3_	120°	***K***_*d*_ (Joint 3)	0.009
*l*_3_	0.14 m	$\dot{\theta }\left(0\right)$	[90,0,0,0] deg/s	*β*_4_	135°		

### 4.2 Control of the configuration

In this section, we discuss the simulation results obtained from different controlling methods. First, *θ*_1_ is a key parameter of the behavior since it is the squirrel’s head direction and also directly connected with the distance of *DR* in the control algorithm.

**FIGURE 8:**
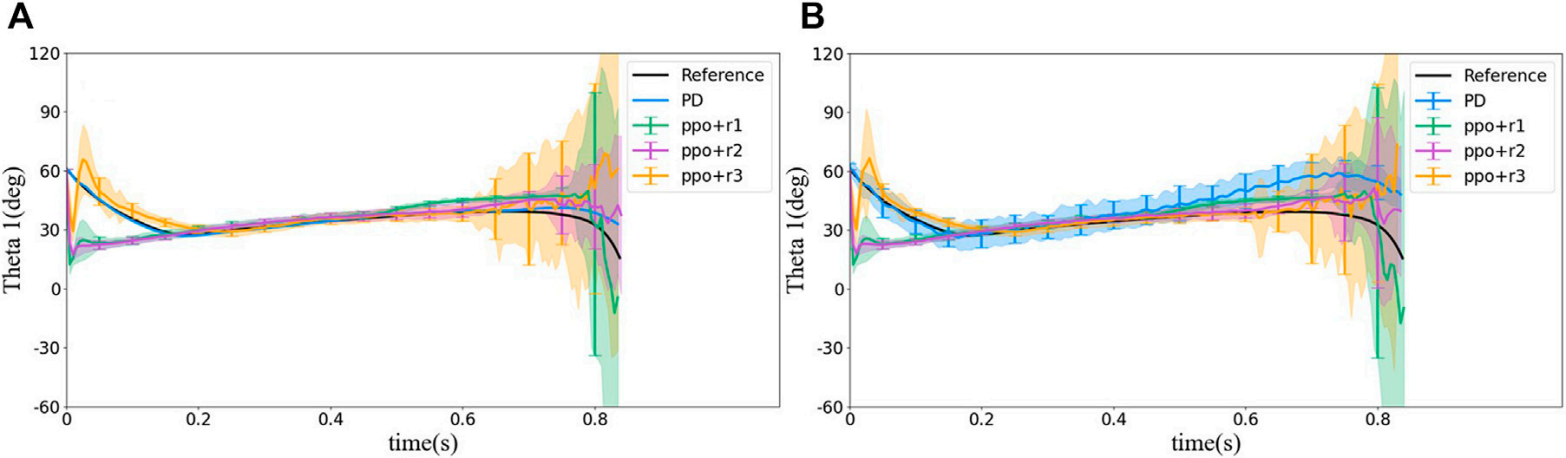
Statistical results of *θ*_1_ simulated using different control methods. **(A)** Without noise and **(B)** with noise. The colored lines show the average with the same colored area as a standard deviation. We sample the standard deviation on some points shown as the vertical bars. The same change applies to all Figures 8–12.

Simulated squirrel’s head orientation (*θ*_1_ values) using different control methods are shown in Figure 8. The reference value of *θ*_1_ (black line) has the following features. The head motion *θ*_1_ can be divided into three parts: 1) quickly turns the head to the orientation to be able to see the landing point (0 s—0.2 s in Figure 7). In this part, the squirrel adjusts its configuration from its initial configuration to the one such that it faces and can observe the landing point. 2) A slow increase in time (0.2 s—0.7 s in Figure 7), and this is the main part of the flight in which the squirrel locks its head toward the landing spot to maintain its visibility. The slow rotation of the head is due to its motion along the projectile trajectory. 3) Rapid decrease in time near landing (0.7 s—0.8375 s in Figure 7). In this part, the squirrel needs to quickly adjust its head orientation near the landing point to maintain its head locking toward the landing point near the end of the projectile trajectory.

**FIGURE 9:**
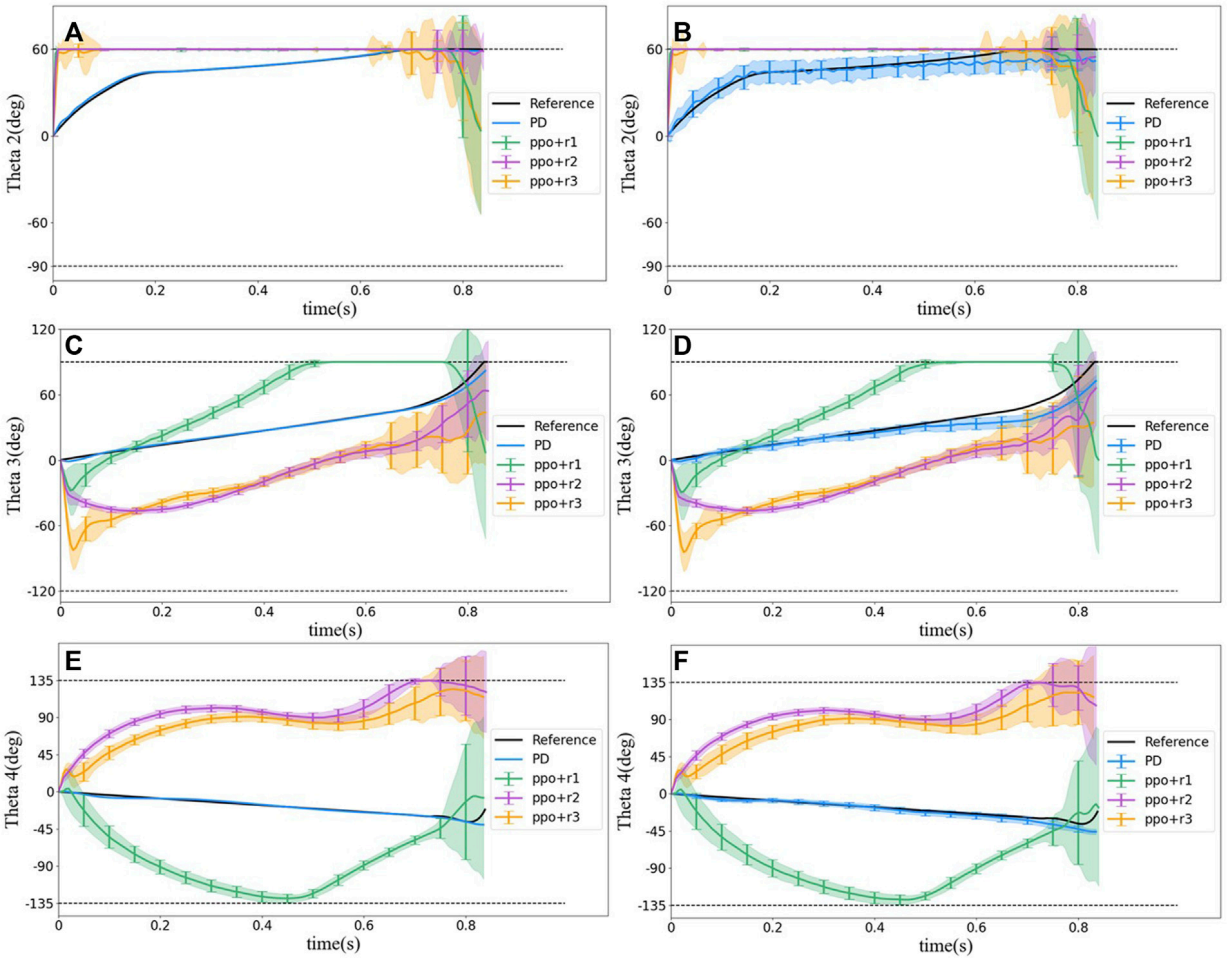
Statistical results of motion behavior of joint 2, 3, and 4 for different control methods. **(A)** Angle between bodies 1 and 2 (*θ*_2_) without noise. **(B)** Angle between bodies 1 and 2 (*θ*_2_) with noise. **(C)** Angle between bodies 2 and 3 (*θ*_3_) without noise. **(D)** Angle between bodies 2 and 3 (*θ*_3_) with noise. **(E)** Angle between bodies 3 and 4 (*θ*_4_) without noise. **(F)** Angle between bodies 3 and 4 (*θ*_4_) without noise.

The PD controller provides almost the same motion trajectory of *θ*_1_ as the pre-planned reference, but *θ*_1_ from PPO with *r*_1_ and *r*_2_ has a shorter part 1 (namely, reaching the locking posture much faster) and a longer part 2, that is because at part 2, the distance of *DR* is close to zero, and thus the agent gets a higher reward. However, a shorter part 1 may result in a more rapid change of the configuration, for example, a higher angular acceleration in each joint, which may exceed the system’s power limit. The reward *r*_3_ is set to extend part 1, but as shown in Figure 8, *θ*_1_ does not follow the reference at the beginning, instead, it decreases quickly as in PPO with reward *r*_1_ and *r*_2_ and increases to target *θ*_1_ soon. Therefore, *r*_3_ did not limit the sudden decrease at the beginning. On the contrary, extra motion is introduced. It should be emphasized that the PD controller tracked the reference trajectory better because it is designed to track the reference, while the RL control does not have any knowledge of the reference. From overall behavior perspective, both controllers achieved the control goal, namely, to quickly turn the head (eyes) toward the landing point after ejected into air and then remained looking at the landing point during the flight until landing.The motion histories of the other joints during the flight are shown in Figure 9. The figure demonstrates that all the joint motions from RL methods are very different from each other and from those of the reference trajectory. For example, in the reference trajectory, the angle between bodies 1 and 2 (i.e., *θ*_2_) move slowly to 60° (i.e., *α*_1_) at about 0.6 s, whereas in RL methods, *θ*_2_ reaches 60° quickly and maintain at the angle for all the time. However, *θ*_1_ values from all methods including the reference trajectory are the same. This phenomenon emphasizes that the reference trajectory may not be a naturally optimal solution although it was calculated from an optimization problem.

### 4.3 Control of the distance of *DR*

**FIGURE 10:**
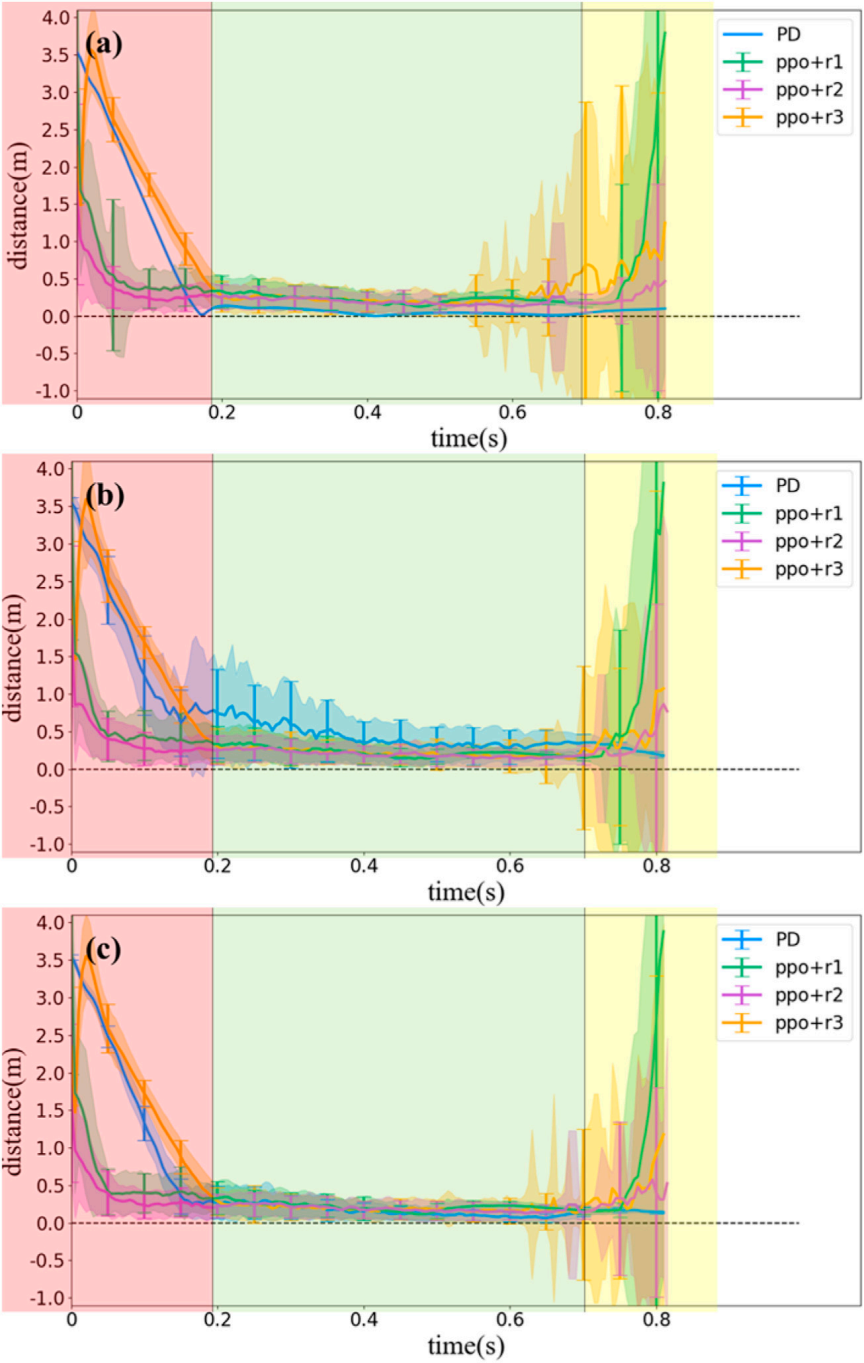
Statistical results of *DR* distance values simulated with different control methods. **(A)** Without noise, **(B)** with noise (0.05*A*), and **(C)** with noise (0.02*A*) (*A* is the maximal measuring range of an input variable).

**FIGURE 11:**
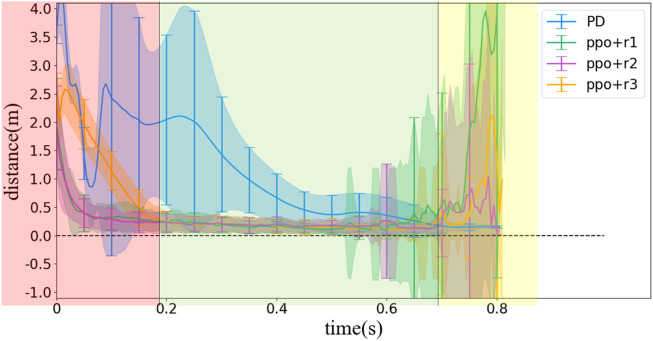
Statistical results of *DR* distance values simulated with different control methods and random initial poses (without noise).

The distance between *D* and *R* is an important and straightforward index to evaluate a control strategy of locking the head toward the landing point. Considering that the distance values of *DR* ranges from 0 to infinity, any distance values over 10 m were recorded as 10 m in our simulation. The value of the *DR* distance for different control strategies is shown in Figure 11. We divide the flight period into three phases as defined in Section 4.2. The first phase lasts about 0.2 s, and the *DR* distance decreases quickly to near zero, while the second phase lasts about 0.5 s, and the main purpose of the strategy transforms to maintaining the *DR* distance near zero. The third phase lasts about 0.13 s, and the main purpose is the same as the second phase but requiring a more rapid rotation of head. In Figure 10, we use red, green, and yellow background to distinguish the three phases.

Figures 10A, B demonstrate some results about the accuracy of the different strategies. First, the PD controller performs better than the RL method under ideal situation (i.e., without noise) but becomes difficult after introducing noise. When random noise is applied as in the reality, the convergence speed of the *DR* distance using the PD controller obviously decreases, while the standard deviation increases, suggesting that the pose at the same time in different ejections varies from each other due to the random noise. However, if the standard deviation of the Gaussian distribution of the noise decreases from 0.05*A* to 0.02*A*, where *A* is the maximal measuring range of an input variable, the PD controller could still maintain the performance better than the RL method, as shown in Figure 10C, suggesting that the PD controller also has certain robustness to noise, but not as robust as the RL method.

Second, all the three RL methods are not so sensitive to the noise as the PD controller, but their variation (with respect to random noise) is more obvious near the landing time. We infer the reason would be that, as the system moves near the landing time, the motion is in the part 3 region, as we have discussed in Section 4.2. However, as the vast majority of the flight is in part 2, the RL method may have learned the strategy that weighs part 2 performance much more than the part 3 performance and is more sensitive when the system changes more frequently.

Third, among the three one-step reward designs, the PPO method with *r*_2_ is the most robust one. There are two aspects for the reason. The first is that the *l*_2_-norm provides more penalty than the exponent of the *l*_2_-norm when $\dot{\theta }$ and ***τ*** become too large. The second is that as the set of *D*_*f*_ in the reward cannot smooth the motion, as shown in Figures 7, 8, the direct reward of the *DR* distance can lead to a longer part 2, which may contribute to the less joint motion variations.

### 4.4 Control performance with random initial poses

For unexpectedly ejected squirrels, the initial pose is unpredictable, thus the motion should be random in joint space. In this section, we also test the performance of different methods with random initial poses which satisfy: 1) *θ*_1_ = 61°; 2) − *β*_*i*_ ≤ *θ*_*i*_ ≤ *α*_*i*_ for *i* = 2, 3, 4; and 3) all bodies should stay above the platform. Since the joint initial motions are random, we only record the *DR* distance to evaluate the performances. Figure 11 illustrates the results. Compared with Figure 10A, we find that the PD controller cannot deal with the random initial conditions well, and that is because the ejection is unexpected and it is impossible to calculate a reference trajectory for each random initial pose, thus we can only use the same reference trajectory, which influences the calculation of error ***ɛ*** for the PD controller. On the other hand, the PPO method keeps almost the same performance with random initial poses, which suggests the robustness of the reinforcement learning-based control strategy.

### 4.5 Control performance with slightly changed models

In reality, the physical properties of a squirrel (i.e., mass and size parameters) are changing over time, but the squirrel can always lock its head/eyes toward the landing point while body tumbling in air. Therefore, it is also important to test the robustness of the strategy provided by the PD controller/RL method. In this section, we randomly changed the parameters of our multilink model, i.e., mass/length/width of each body, by ± 5%/10%/15%/20% without tuning the PD control law or re-training the RL control policy and then observed the corresponding performance of the controllers.

**FIGURE 12:**
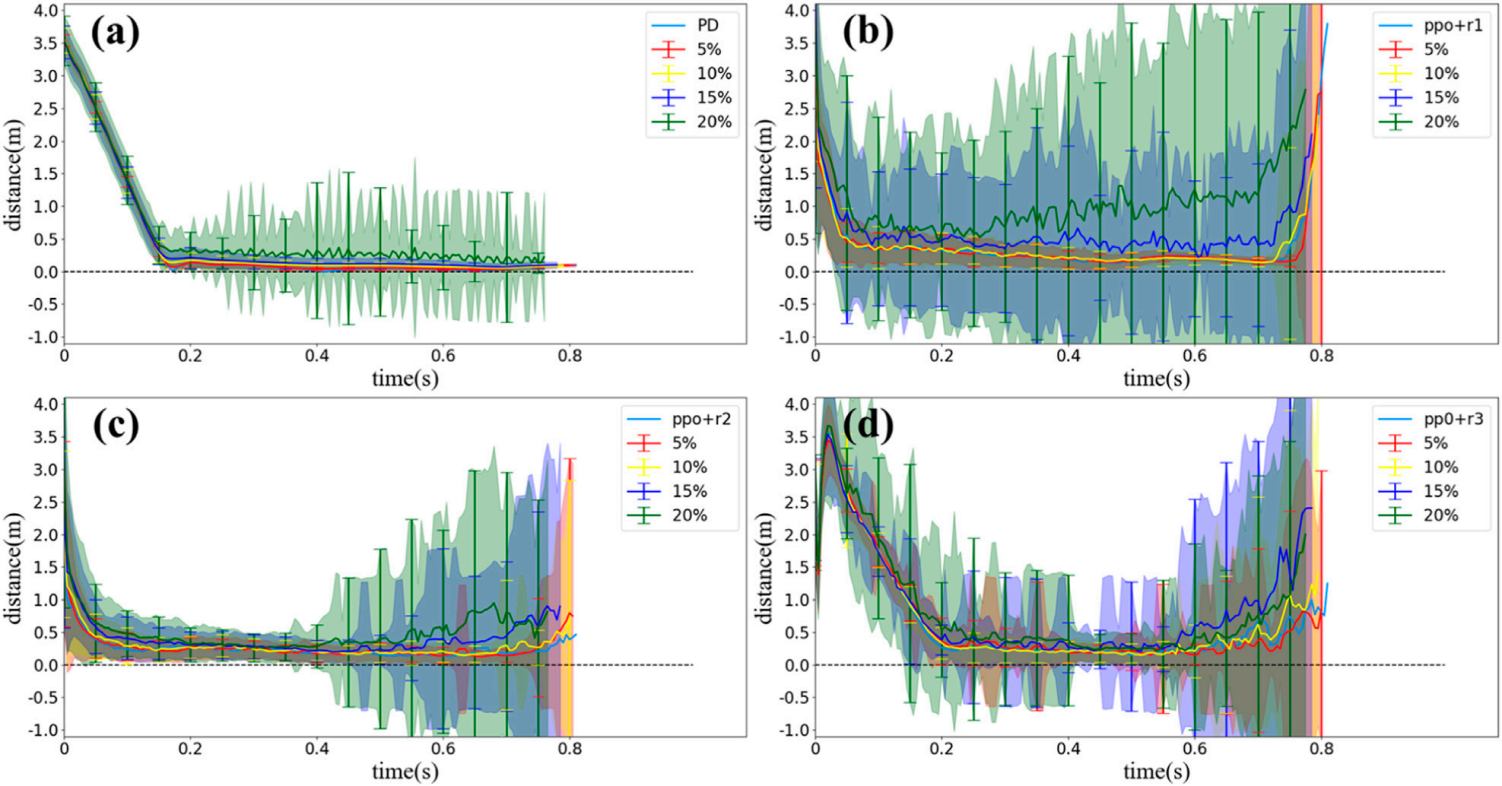
Statistical results of *DR* distance values simulated with different control methods and model errors of 5%–20% (without noise). **(A)** PD controller, **(B)** PPO method with *r*_1_, **(C)** PPO method with *r*_2_, and **(D)** PPO method with *r*_3_.

The results are shown in Figure 12A. For the PD controller, Figures 12A illustrates that the PD controller is robust to model errors by 15%. When the model errors reach 20%, the standard deviation of *DR* distance grows significantly, which means that the motion varies in each simulation. For the RL method, similar results are shown in Figures 12B–D; 10% of model errors does not influence the *DR* distance, but 15% and 20% of model errors can cause uncertainty in different stage of motions. However, in the sense of average, both the PD controller and RL method are robust to the model error even by 20% (except for the PPO method with *r*_1_, but it can still maintain the average under 15% errors).

## 5 Conclusion and future work

In this work, we studied the dynamics and control of a squirrel’s amazing capability of maintaining its head facing the landing spot, while its other body segments tumbling in air after it is unexpectedly ejected into air from any initial pose. To understand the dynamics and explain the observed real squirrel’s behavior, we developed a simplified 2D multibody dynamics model with body segment collision constraints of a squirrel and applied two very different control methods to reproduce the observed squirrel behavior. The first control method is to plan a reference motion trajectory first, representing the squirrel motion behavior and then apply a PD feedback controller to track the planned reference trajectory. The second control method is to use a reinforcement learning process to train a deep neuron-network-based control policy to achieve the squirrel motion behavior. In both control methods, random noise (white noise) is added to the sensed feedback motion data to make the simulated situation closer to the reality. Simulation results demonstrated that both methods successfully achieved the expected control goal of quickly turning the head toward the landing point and then locking the head toward the landing spot during the flight phase. Comparing the two control methods, the RL method performs better in terms of closer to expected behavior and robustness against sensor errors. However, the RL method shows more variant joint motions with respect to noisy input data near the landing time, but these variations are all acceptable because they all achieved the targeted head motion behavior. Another main advantage of the RL method is that we do not need to plan a reference trajectory first, and thus the method would suit more to the natural environment and lead to more natural outcome.

The future work especially focuses on the following aspects: 1) further development of the one-step reward setting for the RL process. Current reward settings still converge to local optimal and cannot stretch the body enough. 2) Expand the dynamics model to the 3D space. To achieve this expansion, the segmentation of the motion and multi-layer control are necessary. Another future research direction of 3D motion is gait analysis, which is to reveal repetitive motion pattern of body segments including legs and tail. The research about locomotion and gait analysis has been explored and studied in legged or snake-like animals or robots (Ostrowski and Burdick, 1998; Guo et al., 2018). Specific body relative motion gaits may exist in squirrel locomotion while in the air, especially the tail motion (Fukushima et al., 2021), but we need to further explore the locomotion in real squirrels and apply the gaits in a more rapid duration. 3) We should try establishing a larger dataset of real squirrel motion behavior. This will support us to obtain more scientific understanding of the observed squirrel behavior and develop better control design for squirrel-like challenging operations of bio-inspired robots or other autonomous systems.

## Data Availability

The original contributions presented in the study are included in the article/Supplementary Material; further inquiries can be directed to the corresponding author.
